# Emergency department urine culture stewardship and downstream outcomes: a covariate-adjusted biweekly interrupted time series analysis

**DOI:** 10.1017/ash.2026.10758

**Published:** 2026-06-29

**Authors:** James Howard Acuff, Ashwini Gotimukul, Ganesh Srinivasan Krishnamurthi, Todd Lasco, Armando Rafael Cecilio Leon Silva, D’Feau Jia Lieu, Kady Phe, Sabra L. Shay, Alfonso Francisco Siu, Nicholas Teran, Mayar Al Mohajer

**Affiliations:** 1 Baylor College of Medicine, USA; 2 Baylor St Luke’s Medical Center, USA; 3 Premier Inc, USA; 4 University of Oxford, UK

## Abstract

**Background:**

Emergency department (ED) urine culture stewardship may reduce low-value testing, but downstream effects on antibiotic use and postdischarge utilization remain uncertain.

**Methods:**

We evaluated an intervention in which an infectious diseases physician and microbiologist reviewed pyuric urinalysis-with-reflex encounters, assessed culture indications, and contacted clinicians to recommend cancellation when absent. Adult ED encounters from February–July 2025 to September 2025-February 2026 were included; August was washout. Outcomes were urinalysis with reflex within 24 hours, urine culture within 24 hours, antibiotic days of therapy (DOT) per 100 patient-days, length of stay (LOS), and 30-day ED revisit among ED discharges. We fit biweekly interrupted time series models.

**Results:**

Among 17,621 encounters, the intervention was associated with lower postintervention slopes for urinalysis with reflex within 24 hours (−0.5 percentage points per biweekly period; 95% CI, −0.9 to −0.1) and urine culture within 24 hours (−0.2 percentage points; 95% CI, −0.4 to −0.0). DOT showed no sustained change (postintervention slope change, 0.00; 95% CI, −0.23 to 0.24). LOS showed a lower postintervention slope (−0.05 d; 95% CI, −0.08 to −0.02). Thirty-day ED revisit showed a higher postintervention slope (0.8 percentage points; 95% CI, 0.3 to 1.3).

**Conclusions:**

The intervention reduced urinary testing but did not reduce antibiotic DOT and was associated with increased 30-day ED revisit. Diagnostic stewardship in the ED may need to be paired with antimicrobial stewardship and prospective safety monitoring.

## Introduction

Urine testing is common in emergency departments (EDs), where clinicians frequently evaluate nonspecific symptoms under time pressure and with incomplete initial data. Yet positive urinalysis or urine culture results are often nonspecific and may identify asymptomatic bacteriuria rather than true urinary tract infection, exposing patients to unnecessary antibiotic treatment and downstream harms.^1,2^ In ED populations, urine testing itself has been associated with inappropriate antibiotic prescribing and longer length of stay, suggesting that diagnostic decisions made early in the encounter can shape both treatment and throughput.^3^

Diagnostic stewardship seeks to improve how tests are ordered, processed, and reported so that microbiologic data more accurately support clinical decision-making.^4,5^ For urine cultures, recommended strategies include restricting culture processing to patients with compatible symptoms or urinalysis findings, embedding decision support, and using laboratory workflows that reduce low-value cultures.^4,5^ Several ED- and hospital-based interventions have shown that reflex or 2-step urine culture approaches can reduce culture volume and improve diagnostic efficiency.^6–9^ However, whether these strategies consistently translate into lower antibiotic exposure or unchanged postdischarge utilization remains uncertain, particularly when stewardship occurs after clinicians have already decided to send urine or begin empiric therapy.^8,9^

We evaluated an intervention in which an infectious diseases provider and microbiologist reviewed urinalysis-with-reflex-to-culture orders with pyuria, assessed concordance between charted symptoms and guideline-based indications, and directly contacted clinicians when culture cancellation was appropriate. We hypothesized that this intervention would reduce urine culture use and would also influence upstream ordering behavior, reflected by lower urinalysis-with-reflex use, but that diagnostic stewardship alone might be insufficient to reduce antibiotic use if empiric treatment decisions continued independent of culture review. We also sought to evaluate balancing outcomes, including length of stay and 30-day ED revisit among ED discharges.

## Methods

We conducted a quasi-experimental interrupted time series study at the study-site ED using encounter-level data from a curated operational stewardship data set. The analytic preintervention period was February 1 through July 31, 2025, and the postintervention period was September 1, 2025, through February 28, 2026; August 2025 was excluded as washout. During the study period, clinicians generally ordered urinalysis with automated reflex to culture, and reflex culture was triggered when urinalysis showed ≥10 urine white blood cells. Clinicians could order urine culture without reflex for pregnancy, neutropenia with urinary symptoms, renal transplantation within 3 months, or planned urologic procedures; patients with HIV infection or active malignancy without neutropenia were not separately exempted and followed the standard reflex pathway. The intervention consisted of infectious diseases physician–microbiologist review of pyuric urinalysis-with-reflex-to-culture encounters, assessment of whether charted symptoms supported guideline-concordant culture indications based on IDSA and expert diagnostic stewardship recommendations,^1,5^ and direct outreach to the ordering clinician to cancel cultures when an appropriate indication was not present.

Eligible encounters occurred at the study site, involved adults 18 years or older, and remained after application of the source exclusion flag. Excluded encounters were those flagged for recent *Clostridioides difficile* infection (14 d before to 3 d after admission), receipt of non-UTI antibiotics within 3 days of admission, a positive nonurinary culture within 3 days of admission, or urine culture results reported only in comments rather than standard A/N format. All urine collection types captured in the data set, including voided and catheter-associated specimens, were included. Data were deduplicated by encounter identifier before analysis. Formal process measures were not prospectively recorded; based on routine workflow, reviewers generally assessed approximately 10–12 cultures per day and contacted clinicians regarding approximately 0–4 potential cancellations or other interventions per day. The Baylor College of Medicine Institutional Review Board approved the study.

Outcomes were urinalysis with reflex within 24 hours, urine culture within 24 hours, antibiotic days of therapy (DOT) per 100 patient-days, length of stay, and 30-day ED revisit among ED discharges. Because indication-specific prescribing was unavailable, DOT captured all antibiotics administered during the encounter rather than urinary-directed therapy alone. For descriptive comparisons, Table 1 used the full pre and postintervention encounter windows. For interrupted time series analyses, outcomes were summarized in complete 14-day periods. We standardized each biweekly outcome to the overall study-site case mix and fit segmented regression models with Newey-West standard errors. Models adjusted for age, sex, ED disposition, and systemic inflammatory response syndrome category; variables with only 1 observed level in an outcome-specific subset were automatically dropped from that standardized model. Primary interrupted time series parameters were preintervention trend, immediate level change, and postintervention slope change.

**Table 1.** Study characteristics of adult emergency department encounters at the study site overall and by study period

Characteristic	Overall N = 17,621	Preintervention N = 9,014	Postintervention N = 8,607	*P* value
Age, y	54.0 (18.4)	54.0 (18.3)	53.9 (18.5)	.625
**Sex**	–	–	–	<.001
Female	9,318 (53%)	4,893 (54%)	4,425 (51%)	–
Male	8,294 (47%)	4,114 (46%)	4,180 (49%)	–
Missing/unknown	9 (<0.1%)	7 (<0.1%)	2 (<0.1%)	–
**ED disposition**	–	–	–	.236
Admitted to ICU	224 (1.3%)	116 (1.3%)	108 (1.3%)	–
Admitted to inpatient unit	5,798 (33%)	2,913 (32%)	2,885 (34%)	–
Discharged from ED	11,599 (66%)	5,985 (66%)	5,614 (65%)	–
**SIRS category**	–	–	–	.078
0 criteria	6,903 (39%)	3,487 (39%)	3,416 (40%)	–
1 criterion	6,290 (36%)	3,202 (36%)	3,088 (36%)	–
≥2 criteria	4,359 (25%)	2,295 (25%)	2,064 (24%)	–
Missing	69 (0.4%)	30 (0.3%)	39 (0.5%)	–
Length of stay, *d*	0.32 (0.16, 2.09)	0.31 (0.16, 2.02)	0.33 (0.16, 2.15)	.067

Note. Mean (SD), n (%), or median (IQR). Adult nonexcluded study-site encounters only. Table 1 uses the full preintervention (February 1-July 31, 2025) and postintervention (September 1, 2025–February 28, 2026) encounter windows. August 2025 was excluded as the washout month. P values compare pre versus postintervention encounters at the study site (t test for age, Wilcoxon rank-sum test for length of stay, and χ^2^ tests for categorical variables). This table is descriptive and unadjusted. ED, emergency department; IQR, interquartile range; LOS, length of stay; SIRS, systemic inflammatory response syndrome.

## Results

Among 17,621 adult, nonexcluded study-site ED encounters included in the full analytic window, 9,014 occurred during the preintervention period, and 8,607 occurred during the postintervention period (Table 1). Mean age was similar across periods (54.0 vs 53.9 yr), whereas sex distribution differed modestly (female 54% vs 51%; *P* < .001). ED disposition and systemic inflammatory response syndrome category were similar across periods.

**Figure 1.**
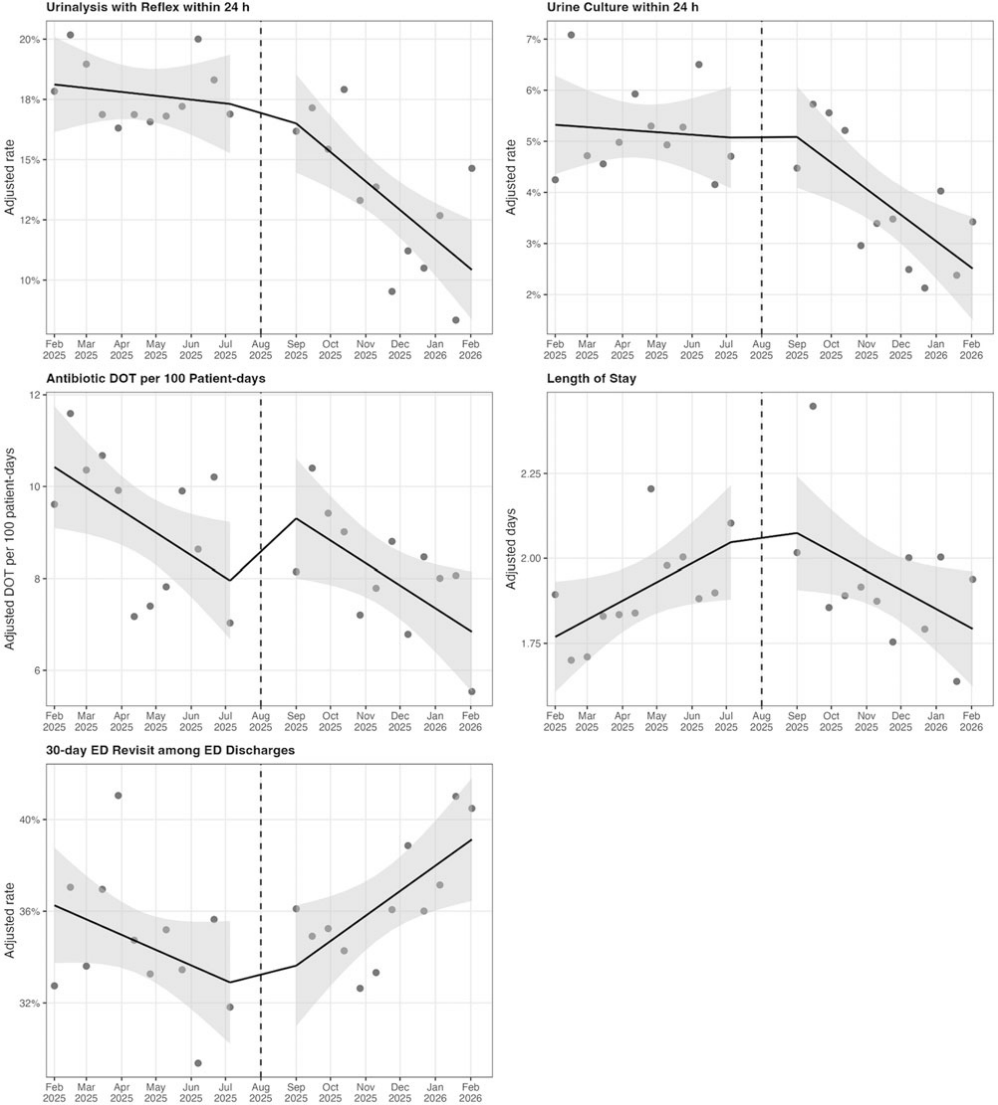
Biweekly interrupted time series of covariate-adjusted study outcomes at the study site. Gray points are covariate-adjusted biweekly values. Black lines are fitted segmented regression lines. Shaded bands are model-based 95% confidence intervals. The dashed line marks the August 2025 washout/intervention month. Outcomes were standardized to the overall study-site case mix before segmented regression. Models were adjusted for age, sex, ED disposition, and systemic inflammatory response syndrome category; variables with only 1 observed level in a given outcome-specific subset were automatically dropped from that standardized model. Abbreviations: DOT, days of therapy; ED, emergency department; LOS, length of stay; SIRS, systemic inflammatory response syndrome.

In covariate-adjusted biweekly interrupted time series analyses, no significant immediate level change was observed for urinalysis with reflex within 24 hours, urine culture within 24 hours, antibiotic DOT per 100 patient-days, length of stay, or 30-day ED revisit among ED discharges (Table 2; Figure 1). However, the intervention was associated with a lower postintervention slope for urinalysis with reflex within 24 hours (−0.5 percentage points per biweekly period; 95% confidence interval [CI], −0.9 to −0.1; *P* = .028) and for urine culture within 24 hours (−0.2 percentage points per biweekly period; 95% CI, −0.4 to −0.0; *P* = .031). These findings indicate gradual, rather than abrupt, reductions in urinary testing after implementation.

**Table 2.** Covariate-adjusted biweekly interrupted time series analysis results

Outcome	ITS term	Estimate	95% CI	*P* value
Urinalysis with reflex within 24 h	Preintervention trend	−0.1 pp	(−0.3, 0.2)	.525
Urinalysis with reflex within 24 h	Immediate level change	−0.3 pp	(−2.9, 2.4)	.843
Urinalysis with reflex within 24 h	Postintervention slope change	−0.5 pp	(−0.9, −0.1)	.028
Urine culture within 24 h	Preintervention trend	−0.0 pp	(−0.1, 0.1)	.677
Urine culture within 24 h	Immediate level change	0.2 pp	(−1.0, 1.5)	.678
Urine culture within 24 h	Postintervention slope change	−0.2 pp	(−0.4, −0.0)	.031
Antibiotic DOT per 100 patient-days	Preintervention trend	−0.22	(−0.40, −0.04)	.017
Antibiotic DOT per 100 patient-days	Immediate level change	1.57	(−0.27, 3.41)	.089
Antibiotic DOT per 100 patient-days	Postintervention slope change	0.00	(−0.23, 0.24)	.996
Length of stay, *d*	Preintervention trend	0.03	(0.01, 0.04)	.004
Length of stay, *d*	Immediate level change	0.05	(−0.17, 0.28)	.632
Length of stay, *d*	Postintervention slope change	−0.05	(−0.08, −0.02)	.002
30-day ED revisit among ED discharges	Preintervention trend	−0.3 pp	(−0.7, 0.1)	.105
30-day ED revisit among ED discharges	Immediate level change	0.2 pp	(−3.4, 3.9)	.897
30-day ED revisit among ED discharges	Postintervention slope change	0.8 pp	(0.3, 1.3)	.004

Note. Covariate-adjusted biweekly interrupted time series analysis at the study site. Biweekly outcomes were standardized to the overall study-site case mix before segmented regression. Table 2 and Figure 1 use complete 14-day biweekly bins only; Table 1 uses the full pre/post encounter windows. August 2025 was excluded as the washout month. Models were adjusted for age, sex, ED disposition, and systemic inflammatory response syndrome category; variables with only 1 observed level in a given outcome-specific subset were automatically dropped from that standardized model. Binary outcomes are shown as percentage-point (pp) changes. CI, confidence interval; DOT, days of therapy; ED, emergency department; LOS, length of stay; SIRS, systemic inflammatory response syndrome.

Antibiotic DOT per 100 patient-days demonstrated a declining preintervention trend (−0.22 per biweekly period; 95% CI, −0.40 to −0.04; *P* = .017), but there was no intervention-associated immediate level change (1.57; 95% CI, −0.27 to 3.41; *P* = .089) or postintervention slope change (0.00; 95% CI, −0.23 to 0.24; *P* = .996). Length of stay showed no immediate level change but did show a lower postintervention slope (−0.05 d per biweekly period; 95% CI, −0.08 to −0.02; *P* = .002).

In contrast, 30-day ED revisit among ED discharges showed a positive postintervention slope change (0.8 percentage points per biweekly period; 95% CI, 0.3 to 1.3; *P* = .004) without an immediate level change. In the adjusted biweekly series, revisit values were in the low-to-mid 30% range during the earlier postintervention periods and rose into the upper 30% to approximately 40% range by the final biweekly periods (Figure 1). Overall, the intervention was associated with less urinary testing, no measurable reduction in antibiotic exposure, and a higher postintervention revisit trend that warrants further evaluation as a potential safety signal.

## Discussion

This study shows that an ED urine culture stewardship intervention built around infectious diseases physician–microbiologist review and culture cancellation was associated with gradual reductions in both urine culture use and urinalysis-with-reflex ordering, but not with a sustained reduction in overall antibiotic DOT. At the same time, 30-day ED revisit among ED discharges increased over the postintervention period. Taken together, these findings suggest that diagnostic stewardship reduced urinary testing, but the intervention may not have been sufficient to change downstream antibiotic prescribing and may have been accompanied by a potential safety signal requiring further evaluation rather than reassurance.

The lower postintervention urine culture slope is directionally consistent with prior ED and hospital-based urine culture stewardship studies using 2-step ordering, reflex culture strategies, or other diagnostic stewardship interventions.^6–9^ Unlike those automated approaches, our intervention relied on manual infectious diseases physician–microbiologist review and direct clinician outreach rather than a fully automated laboratory hard stop. The concurrent decline in urinalysis-with-reflex ordering is notable because the intervention did not directly target initial urine ordering. One plausible explanation is spillover to upstream clinician ordering, whereby repeated feedback made clinicians more selective about when to initiate urine testing. However, because prescriber-level behavior and formal process metrics were not measured, this finding should be interpreted as compatible with, rather than definitive proof of, clinician behavior change and more broadly consistent with diagnostic stewardship principles.^4,5^

The absence of a sustained DOT reduction is equally informative. Because DOT captured all antibiotics administered during the encounter rather than only urinary-directed therapy, any effect on UTI-specific prescribing may have been diluted. In addition, diagnostic stewardship can occur after empiric treatment has already started, so fewer cultures may not translate into immediate changes in prescribing momentum. This interpretation is consistent with prior work suggesting that urine diagnostic stewardship has its largest antimicrobial impact when paired with direct review of antibiotic initiation, de-escalation, or discontinuation.^8,10–12^ We were not aware of another contemporaneous ED stewardship intervention directed at urinary prescribing during the study period, although secular changes in case mix or prescribing cannot be excluded and may partly explain the preintervention DOT decline.

The 30-day ED revisit finding deserves direct emphasis. We observed no immediate level change, but a progressively rising postintervention slope in a broad postdischarge utilization outcome. Revisit captured any return ED encounter within 30 days among discharged patients and should not be interpreted as UTI-specific recurrence. Although segmented regression estimated the preintervention trajectory and therefore partly accounts for baseline secular drift, seasonal confounding remains plausible because the postintervention period fell entirely within fall and winter months. Historical same-calendar-month data before 2025 were not accessible within the curated operational data set, and reconstruction of an equivalently standardized comparison cohort was not feasible. We did not perform chart review, ICD-code categorization, or time-to-revisit analysis, and we were not prospectively alerted to revisit encounters by ED or hospitalist teams; therefore, we cannot determine whether revisits were urinary, septic, respiratory, or unrelated, and this finding should be interpreted as a hypothesis-generating safety signal rather than evidence of intervention-attributable harm.

Additional limitations include single-center design, residual secular trends, reliance on a curated operational exclusion flag, inclusion of heterogeneous urine specimen types, and absence of formal process metrics such as reviewed orders, cancellation recommendations, clinician acceptance, and week-to-week intervention counts. Despite these limitations, the study offers a practical message: diagnostic stewardship can reduce downstream urinary testing in the ED, but culture-focused interventions alone may not reduce overall antibiotic exposure and should be paired with antimicrobial stewardship and prospective safety monitoring.
